# Farnesyl Diphosphate Synthase Inhibitors from *In Silico* Screening

**DOI:** 10.1111/cbdd.12121

**Published:** 2013-05-25

**Authors:** Steffen Lindert, Wei Zhu, Yi-Liang Liu, Ran Pang, Eric Oldfield, J Andrew McCammon

**Affiliations:** 1 Department of Pharmacology, University of California San DiegoLa Jolla, CA, 92093, USA; 2 NSF Center for Theoretical Biological PhysicsLa Jolla, CA, 92093, USA; 3 Center for Biophysics & Computational Biology, University of Illinois at Urbana-ChampaignUrbana, IL, 61801, USA; 4 School of Molecular and Cellular Biology, University of Illinois at Urbana-ChampaignUrbana, IL, 61801, USA; 5 Department of Chemistry, University of Illinois at Urbana-ChampaignUrbana, IL, 61801, USA; 6 Howard Hughes Medical Institute, University of California San DiegoLa Jolla, CA, 92093, USA; 7 Department of Chemistry & Biochemistry National Biomedical Computation Resource, University of California San DiegoLa Jolla, CA, 92093, USA

**Keywords:** drug discovery, molecular modeling, virtual screening

## Abstract

The relaxed complex scheme is an *in silico* drug screening method that accounts for receptor flexibility using molecular dynamics simulations. Here, we used this approach combined with similarity searches and experimental inhibition assays to identify several low micromolar, non-bisphosphonate inhibitors, bisamidines, of farnesyl diphosphate synthase (FPPS), an enzyme targeted by some anticancer and antimicrobial agents and for the treatment of bone resorption diseases. This novel class of farnesyl diphosphate synthase inhibitors have more drug-like properties than existing bisphosphonate inhibitors, making them interesting pharmaceutical leads.

The enzyme farnesyl diphosphate synthase (FPPS) has been identified as an interesting target for antitumor and anti-infective drug leads. Farnesyl diphosphate synthase is a key enzyme in the mevalonate isoprenoid biosynthesis pathway and is responsible for the condensation of dimethylallyl diphosphate with isopentenyl diphosphate (IPP) to form geranyl diphosphate (GPP) and, subsequently, farnesyl diphosphate (FPP) 1,2. Farnesyl diphosphate is important as a substrate in subsequent steps to synthesize key molecules like cholesterol and ergosterol. Pharmaceutically, FPPS has gained importance in the treatment of malignant bone disease, as inhibiting FPPS blocks excessive bone resorption in osteoclasts by causing apoptosis 3. However, inhibition of FPPS has implications beyond bone disease as preclinical research has shown direct antitumor activity in a variety of human cancers 4,5. Additionally, the isoprenoid biosynthesis pathway is essential for bacterial cell wall biosynthesis as the synthesis of peptidoglycan depends on formation of lipid I and lipid II from undecaprenyl phosphate, an isoprenoid derived from FPP. Farnesyl diphosphate synthase’s role in ergosterol biosynthesis also makes it an interesting target in the search for drug leads against Chagas disease and the leishmaniases, neglected tropical diseases that affect approximately 10 million individuals.^a^

Historically, bisphosphonates were the first FPPS inhibitors identified 6,7 and were developed as bleaching herbicides that block carotenoid biosynthesis. It was then shown that bisphosphonate bone resorption drugs such as alendronate (Fosamax) targeted FPPS, and it appeared 8,9,10 that bisphosphonates might be the only efficient inhibitors of the enzyme. From a pharmaceutical perspective, bisphosphonates have several undesirable features for anti-infective or anticancer drug leads in that they are highly polar as well as being prone to rapid removal from the circulatory system by binding to the bone mineral 11. There has thus been interest in the development of more apolar bisphosphonates and even non-bisphosphonate FPPS inhibitors 12,13,14. In particular, a fragment-based approach identified several non-bisphosphonate FPPS inhibitors that targeted a new, allosteric binding site 3. The pocket is mainly defined by helices C, G, H and a part of helix J, with some residues from the B-C loop, the H-I loop, helix A and the C-terminal loop contributing to a lesser extent. The pocket itself is amphipathic in nature. It has a hydrophobic base and rear side, centered on residues F206, F239, L344, I348 and Y10. The opposite side is polar with several positively charged (K57, R60, K347) as well as polar (N59, T63) residues. These non-bisphosphonate FPPS inhibitors may represent novel anticancer drug leads as they are not expected to bind to bone mineral 11. To build on this work, we carried out a virtual screening study targeting the FPPS allosteric binding site. For this, we used the relaxed complex scheme (RCS), an *in silico* drug screening method that accounts for receptor flexibility using molecular dynamics simulations 15,16,17. A previously reported MD and docking study on FPPS did not target the allosteric site 12. Virtual screens were performed with AutoDock Vina 18 and Glide 19,20 on crystal structure data as well as numerous structures from a FPPS molecular dynamics simulation. A neural network rescoring was performed to optimize the ranking of known inhibitors, and 10 consensus predictions were screened experimentally yielding one hit, which was further improved by a similarity search, yielding three low (1.8–2.5) micromolar leads. To our knowledge, this is the first successful virtual screen into the FPPS allosteric site.

## Methods and Materials

### Crystal structures and structural ensemble from molecular dynamics simulations

We carried out a virtual screen of the FPPS allosteric site using the crystal structures described by Jahnke *et al*. 3. In addition, we carried out a second virtual screen using representative snapshots from an MD simulation of FPPS. The setup for the MD simulation is described in detail in 12. Frames every 20 ps were extracted from the MD trajectories; the frames were aligned using all C_α_ atoms in the protein and subsequently clustered by RMSD using GROMOS++ conformational clustering 21. The chosen RMSD cutoff resulted in 23 clusters that reflected most of the trajectory. The central members of each of these clusters were chosen to represent the protein conformations within the cluster and, thereby, the conformations sampled by the trajectory. The central member of a cluster (also referred to as ‘cluster center’) is the structure that has the lowest pairwise RMSDs to all other members of the cluster.

### Docking and rescoring of known non-bisphosphonate allosteric site inhibitors

To assess the abilities of the docking software, the 12 ligands described in 3 were docked. For those compounds where no crystal structure information was available, the ChemDraw file was converted to PDB format using Open Babel 22. For the AutoDock Vina screens, pdb2pqr 23,24 was used to add hydrogen atoms to the crystal structure receptor. The AutoDock scripts 25 prepare_ligand4.py and prepare_receptor4.py were used to prepare ligand and receptor PDQBT files. A docking grid of size 18.0 Å × 18.0 Å × 18.0 Å, centered on the position of the ligand in the allosteric site, was used for docking. For Glide docking, the ligands were prepared using LigPrep, and the receptors were prepared using the tools provided in the Maestro Protein Preparation Wizard and the Glide Receptor Grid Generation.

For rescoring of AutoDock Vina docked poses, we used the python implementation of NNScore 1.0 in combination with a consensus of the top three scoring networks (12.net, 16.net and 20.net).

### Receiver operating characteristics analysis

A receiver operating characteristics–area under the curve (ROC-AUC) analysis 25 was performed on all known allosteric site crystal structures as well as the 23 MD cluster centers. For this, the eight FPPS allosteric site inhibitors with IC_50_ values <100 μm from 3 were combined with the Schrödinger decoy library [1000 compounds with average molecular mass approximately 400 Da 19,20]. All compounds in the decoy set were assumed to be inactive. Both AutoDock Vina and Glide were then used to dock the 1008 compounds into the allosteric sites of all 32 receptor structures. The compounds were ranked by their AutoDock Vina scores and Glide XP docking scores, and AUC values were calculated from the ROC analysis.

### Virtual screen of NCI diversity set II

The virtual screen was performed using the National Cancer Institute (NCI) diversity set II, a subset of the full NCI compound database. Ligands were prepared using LigPrep, adding missing hydrogen atoms, generating all possible ionization states, as well as tautomers. The final set used for virtual screening contained 1541 compounds. Docking simulations were performed with both AutoDock Vina 18 and Glide 19,20,27. An additional rescoring was performed on the AutoDock Vina results using NNScore. Finally, the individual Glide rankings and NNScore results were combined to form a consensus list of compounds that scored well with both methods.

### Experimental inhibition assay

Human FPPS was expressed and purified and inhibition assays carried out as described previously 14. Briefly, FPPS inhibition assays were carried out using 96-well plates with a 200-μL reaction mixture in each well. The condensation of GPP (100 μm final) and IPP (100 μm final) was monitored at room temperature using a continuous spectrophotometric assay for phosphate-releasing enzymes 28. The reaction buffer contained 50 mm Tris–HCl (pH 7.4), 1 mm MgCl_2_ and 0.01% Triton X100. The compounds investigated were preincubated with enzyme for 30 min at room temperature. The IC_50_ values were obtained by fitting dose–response curve using prism 4.0 (GraphPad Software Inc., La Jolla, CA, USA, www.graphpad.com).

## Results and Discussion

### Docking known non-bisphosphonate inhibitors into the FPPS allosteric site

**Figure 1:**
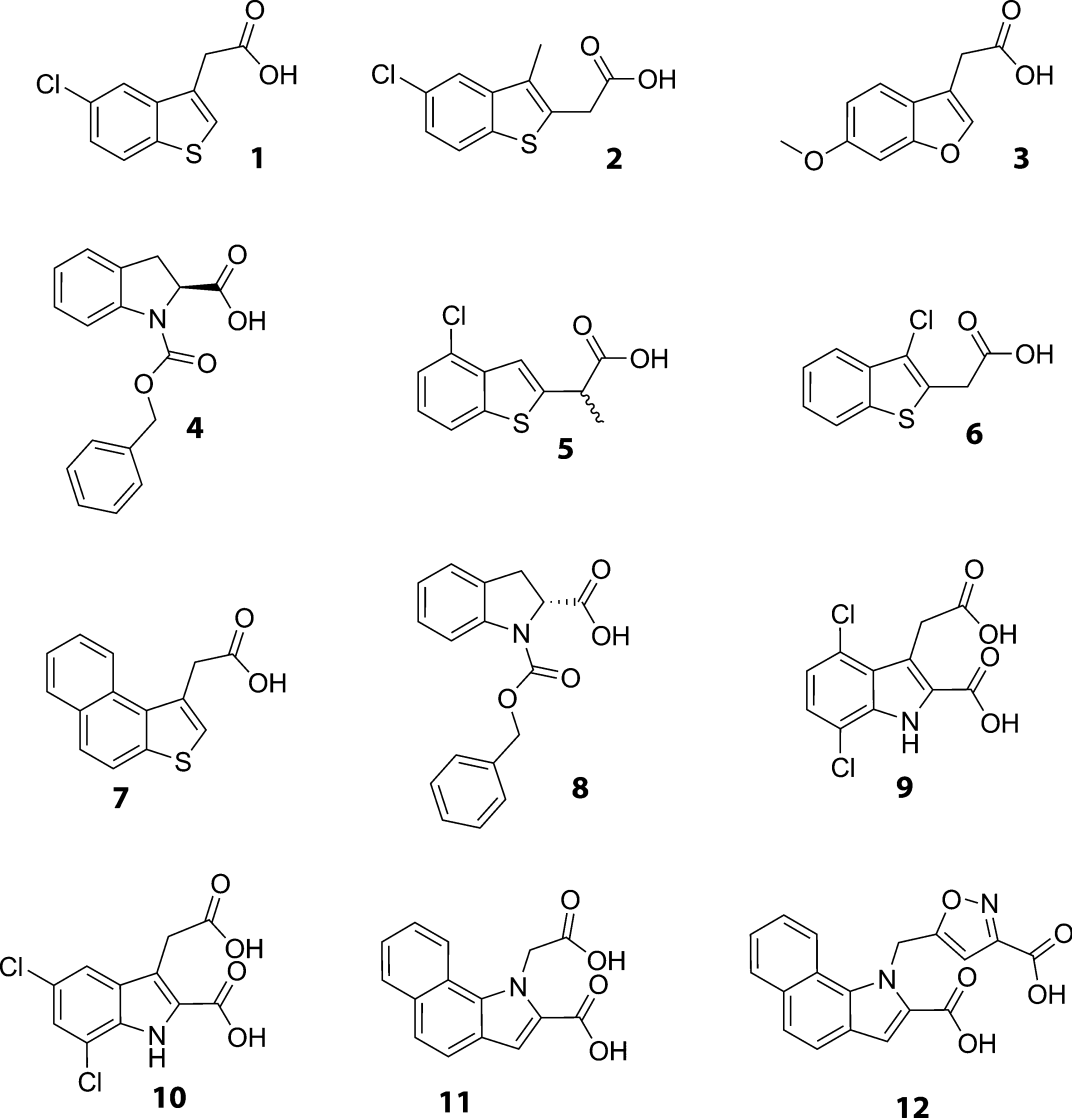
Set of 12 compounds known to bind to the farnesyl diphosphate synthase (FPPS) allosteric site. These ligands were used as positive controls and benchmark compounds to optimize the virtual screens.

For control and benchmarking purposes, the 12 ligands (**1**–**12**, Figure 1) described in 3 were docked using AutoDock Vina 18 and Schrödinger’s Glide 19,20,27. These compounds have IC_50_ values between 80 nm and 500 μm, and all are thought to target a previously unreported allosteric binding site. Compound **11**, the most potent inhibitor with a published structure (PDB-ID 3N6K), has an IC_50_ of 200 nm. No structure was published for compound **12**, the best (80 nm) inhibitor.

**Figure 2:**
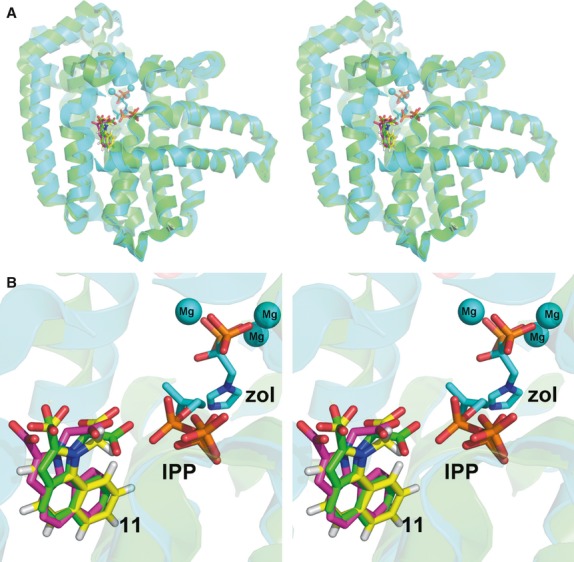
Stereo presentation of docked poses of compound 11 into the farnesyl diphosphate synthase (FPPS) allosteric site (green; PDB ID code 3N6K), superimposed on zoledronate and isopentenyl diphosphate (IPP)-bound structure (cyan; PDB ID code 2F8Z). (A) The RMSD between the crystallographic (green) and docked pose for 11 is 0.8 and 0.6 Å, using AutoDock Vina (purple) and Glide (yellow), respectively. Also shown for reference are zoledronate (in the allylic binding site) and IPP (in the homoallylic binding site; PDB ID code 2F8Z). (B) Expanded view of the ligand binding sites in (A).

The bound pose of compound **11**, as well as the relative binding affinities of the 12 compounds, was then used as positive control to fine-tune the virtual screen parameters. First, compound **11** was docked into the allosteric site of 3N6K using AutoDock Vina. The top scoring model (predicted binding affinity −7.8 kcal/mol) recaptured the published binding pose to within 0.8 Å RMSD (Figure 2). Similarly, Glide correctly found the experimentally determined binding pose of compound **11** (0.6 Å RMSD, Figure 2). This establishes that both AutoDock Vina and Glide can correctly predict bound poses for the FPPS allosteric site which, as is apparent from Figure 2A, B, is distinct from the bisphosphonate (zoledronate) or IPP binding sites.

A much harder task is to computationally predict the relative binding affinities of multiple known binders. To address this question, all 12 compounds were docked into 3N6K and the 23 ensemble structures from MD, using AutoDock Vina. Encouragingly, compound **12** (the most potent compound) scored best, with a predicted binding affinity of −9.3 kcal/mol. AutoDock Vina was not, however, able to properly recapture the relative affinities of the 12 compounds. More specifically, **3** and **4** had high predicted affinity (rank 4 and 2, respectively), while **9** and **10** were only predicted to be weak binders.

We thus next used a neural network approach [NNScore 1.0, 29] to rerank the compounds. NNScore has been developed to characterize the binding affinities of docked protein–ligand complexes by distinguishing between well-docked, high-affinity ligands and well-docked, low-affinity decoy compounds, through neural-network-based rescoring. Testing this approach by rescoring the AutoDock Vina docked poses of the 12 compounds we found that the relative ranking among the compounds (as well as the relative rankings against a large drug database) improved considerably. Now, compounds **9, 10** and **12** were the top three scoring docked compounds, so we then rescored all the AutoDock Vina virtual screen results with NNScore.

All 12 compounds were also docked into the 3N6K crystal structure and the 23 ensemble structures from MD, using Glide. Here, **11** was the top scoring compound (with a predicted binding affinity of −7.5 kcal/mol). Also, Glide identified **9, 10** and **11** as the top three scoring compounds. Given the excellent internal ranking, no rescoring was performed on the Glide docking results.

### Receiver operating characteristics analysis of FPPS structures for enriching known active compounds

**Figure 3:**
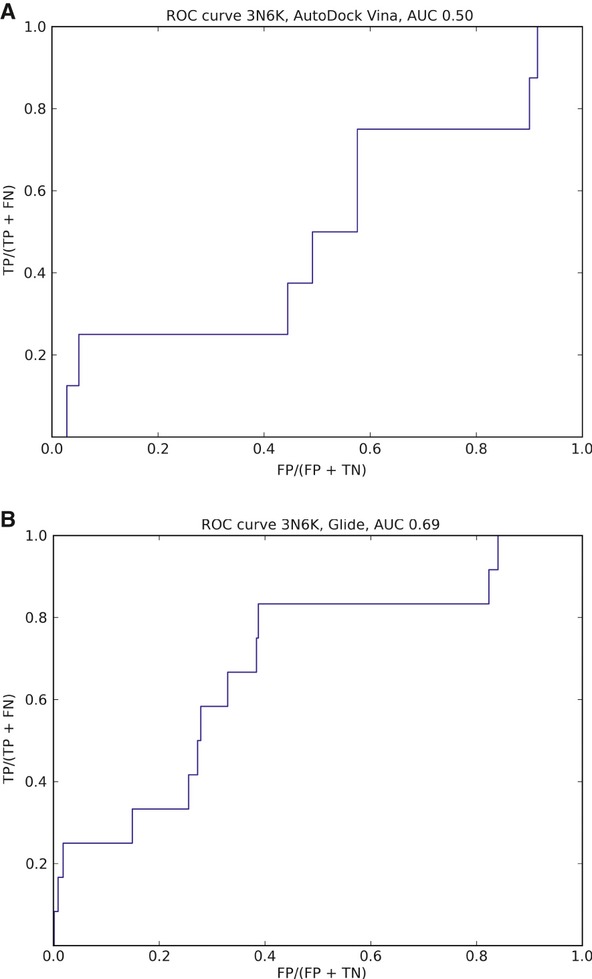
Receiver operating characteristics–area under the curve (ROC-AUC) analysis of 3N6K farnesyl diphosphate synthase (FPPS) structures in terms of enrichment for actives under 100 μm. Compounds 5–12 were used as actives. Docking was performed with (A) AutoDock Vina and (B) Glide.

In addition to the analysis of the ability of the 3N6K crystal structure and the 23 MD cluster centers to rank **5–12** by activity, a ROC-AUC analysis 26 was performed on all crystal structures 3 as well as the 23 MD cluster centers. The highest AUC (0.50) for the AutoDock Vina analysis was obtained with the 3N6K crystal structure, and a number of cluster centers (clusters 23, 13, 7, 17 and 6) also performed quite well in identifying active compounds. Interestingly, the Glide analysis showed a bias toward the crystal structures: the average AUC was 0.65, while the average AUC for the cluster centers was 0.35. The best AUC results were with 3N49 (AUC = 0.76) and 3N6K (AUC = 0.69). Figure 3 shows the ROC curves for 3N6K using both AutoDock Vina and Glide. Based on these results and the ranking analysis described above, we elected to use 3N6K (the structure of the most potent inhibitor) as well as the 23 MD cluster centers for the virtual screen.

### Virtual screen of NCI diversity set II

The 3N6K FPPS crystal structure and the 23 structurally representative snapshots from the FPPS MD simulation were then used as receptors for the RCS docking protocol 15,16,17. Potentially including additional crystal structures might have improved the screening results. This will be the focus of future studies. The data set used in the virtual screen was the NCI diversity set II. The rationale behind using two docking programs (AutoDock Vina and Glide) was that a consensus result would have a better chance of producing good leads. The Glide docking results were ranked according to the predicted docking score, and the AutoDock Vina results were rescored with NNScore since, as discussed previously, this strategy gave the best ranking for the set of known allosteric site inhibitors. A consensus score was used to build up a list of compounds that scored well with both methods. The top 10 compounds from this list had either an NNScore value of >0.71 (the NNScore value for control compound **11**) or a Glide score of <−7.46 kcal/mol (the Glide score for control compound **11**) and were selected for experimental investigation. Upon confirmation of the experimental activity of **13**, a computational similarity search of the entire NCI database was then performed based on **13,** using a Tanimoto index of 90% or higher as the search criterion. The receptors that contributed to the good consensus score of compound **13** were cluster centers 10 (Vina/NNScore) and 18 (Glide). So while the inclusion of all cluster centers into the RCS docking analysis may seem like a drawback in light of the AUC analysis, it was vital for identification of compound **13**. This underscores the power of the RCS method to identify compounds that a screen into the crystal structure alone would not have identified. The docked pose of compound **13** shows several stabilizing interactions such as π-stacking interactions with F251 and hydrogen bonds with N49 and R50. Obtaining a crystal structure is the focus of active ongoing research, so that we decided to not include the docked pose here until confirmed by X-ray crystallography.

### Experimental results

**Figure 4:**
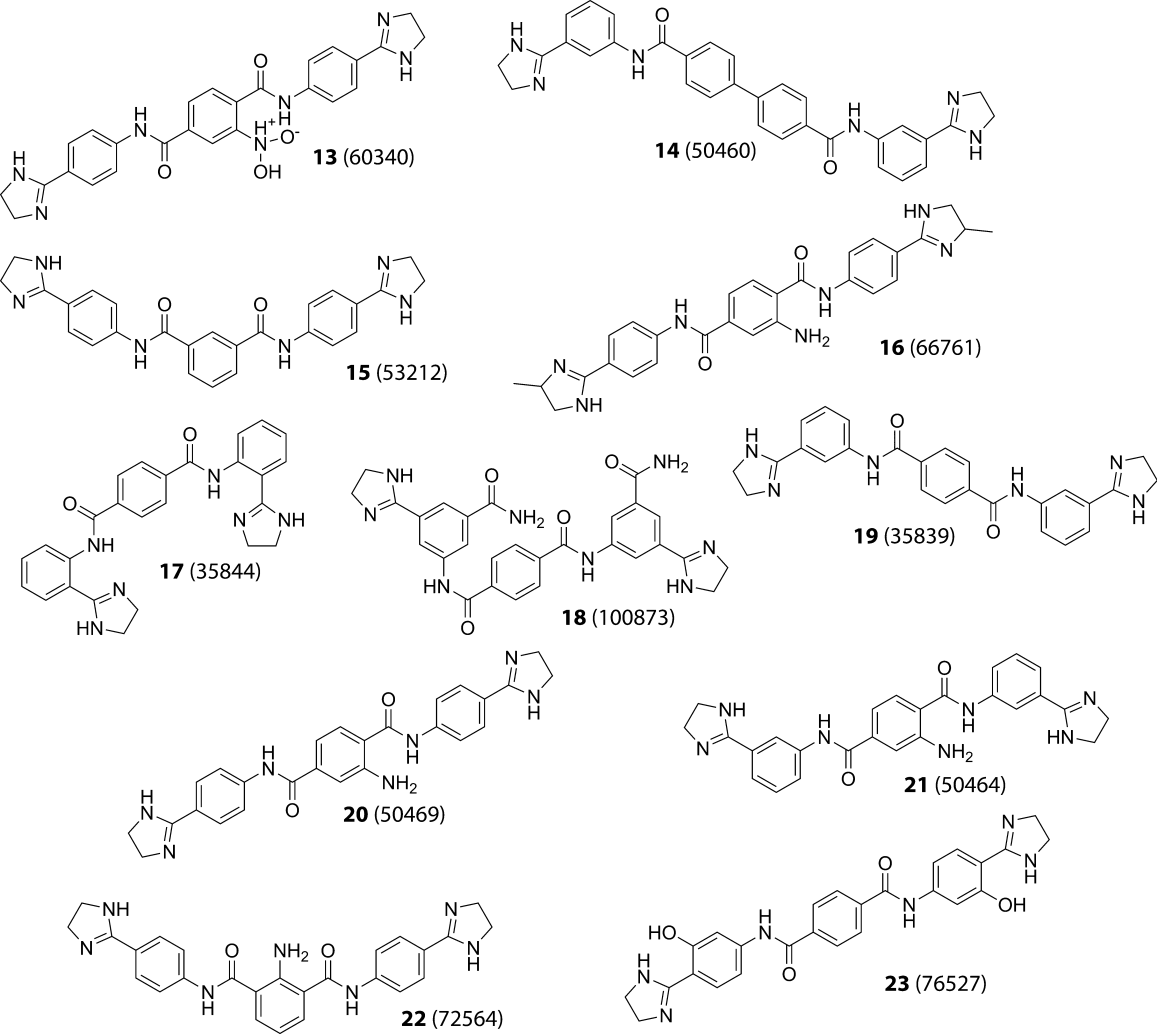
Structures of the bisamidine inhibitors. Shown for convenience (in parentheses) are the National Cancer Institute (NCI) code numbers.

The top 10 compounds identified by the virtual screen were tested experimentally. Compound **13** (Figure 4) was the only experimental hit and had an IC_50_ value of 109 μm. Following the similarity search, additional compounds from the NCI database were screened. Three compounds having IC_50_ values in the approximately 2–3 μm range were identified: **14**, IC_50_ 1.8 μm;**15**, IC_50_ 1.9 μm; and **16**, IC_50_ 2.5 μm. Additionally, there were a number of other compounds found to have IC_50_ values in the low micromolar range: **17**, IC_50_ 7.0 μm;**18**, IC_50_ 10.7 μm;**19**, IC_50_ 13.7 μm;**20**, IC_50_ 20.3 μm;**21**, IC_50_ 21.0 μm;**22**, IC_50_ 22.3 μm; and **23**, IC_50_ 35.0 μm. Table 1 summarizes the experimentally determined IC_50_ results. The search for improved compounds will be an important extension of this work. The most active compound investigated so far is a bisamidine containing a central hydrophobic biphenyl core. Polar substitutions into this central hydrophobic core region abolish activity. The lead compounds have IC_50_ values that are larger than those found with the bisphosphonate zoledronate (IC_50_ = 0.2 μm in this assay). However, as they lack the bisphosphonate feature, they are likely to have longer residence times in plasma, because they will not bind to bone mineral, as well as better cell permeability.

**Table:** Enzyme inhibition results

Compound	Human FPPS IC_50_ (μm)
**13**	109
**14**	1.8
**15**	1.9
**16**	2.5
**17**	7.0
**18**	10.7
**19**	13.7
**20**	20.3
**21**	21.0
**22**	22.3
**23**	35.0

FPPS, farnesyl diphosphate synthase.

## Conclusions

A number of leads for non-bisphophonate FPPS inhibitors have been identified in a RCS virtual screen of the allosteric binding site. The most potent leads, **14–16**, were all bisamidines with IC_50_ values in the approximately 2–3 μm range that also satisfy Lipinski’s rule of five 30 (Suite 2012: QikProp, version 3.5; Schrödinger, LLC, New York, NY, USA, 2012). In other work, we have also found that **14** is also an inhibitor of undecaprenyl diphosphate synthase (from *Staphylococcus aureus*) with an approximately 100 nm IC_50_ 31, opening up the possibility of developing dual FPPS/UPPS inhibitors. How these compounds bind to FPPS remains, however, to be determined, as they are clearly larger than the largest and most potent allosteric site inhibitor, **12**. Other bisamidines are known to be potent antibacterials 32,33, and it has been proposed that some bind to DNA 33, while others inhibit bacterial cell wall biosynthesis 34. The results presented here suggest that in some cases, FPPS inhibition may be another target, with multisite targeting being of particular interest in the context of decreasing the likelihood of the development of drug resistance.

The results of this study also suggest possible avenues of optimization. We propose the following steps that can improve the next generation of virtual screens on the FPPS allosteric site: (i) A RCS approach seemed helpful (given that the best scoring models for compound **13** were reported for docking into cluster centers 10 and 18). In the future, simulations on the actual allosteric site–bound conformation (3N6K) might, however, give an even clearer picture of the dynamics of this binding site. (ii) Future studies should use the previously determined binding affinities as well the affinities reported in this manuscript for a more comprehensive AUC analysis. (iii) Inclusion of more than one crystal structure may improve results as well. (iv) A pocket volume analysis of the allosteric binding site may be able to identify additional structures that can be included in the AUC analysis and, possibly, future screens. (v) Results of future 3N6K simulations should be used to screen for additional druggable hot spots on FPPS: this could be followed up by more virtual screens targeting the identified sites.

## Note

a WHO Parasitic Diseases Leishmaniasis http://www.who.int/vaccine_research/diseases/soa_parasitic/en/index3.html.
